# The Secondary Metabolites Profile in Horse Chestnut Leaves Infested with Horse-Chestnut Leaf Miner

**DOI:** 10.3390/molecules27175471

**Published:** 2022-08-25

**Authors:** Małgorzata Materska, Marzena Pabich, Monika Sachadyn-Król, Agata Konarska, Elżbieta Weryszko-Chmielewska, Barbara Chilczuk, Monika Staszowska-Karkut, Izabella Jackowska, Marta Dmitruk

**Affiliations:** 1 Department of Chemistry, Faculty of Food Science and Biotechnology, University of Life Sciences in Lublin, Akademicka 15, 20-950 Lublin, Poland; 2 Department of Botany and Plant Physiology, University of Life Sciences in Lublin, Akademicka 15, 20-950 Lublin, Poland

**Keywords:** *Aesculus hippocastanum*, *Cameraria ohridella*, phenolic compounds, catechins, anatomy of leaves

## Abstract

Natural defensive substances synthesized by plants that could replace synthetic pesticides in the protection of plants against insect invasions are constantly being sought. The study assessed changes in the qualitative and quantitative composition of secondary metabolites in horse chestnut leaves collected in different locations and differing in the sensitivity of the plant to the invasion by the horse-chestnut leaf miner. An attempt was made to identify compounds that are most responsible for the increased plant resistance to this threat. Additionally, changes in the anatomy of chestnut leaves affected by the pest were presented. It was noticed that the trees differed in the composition of secondary metabolites already in the initial growing season, which should be related to the influence of habitat conditions. The analysis of the profile of the compounds in non-infested and infested horse chestnut leaves revealed a clear response of the plant to the stress factor, i.e., the foraging of the horse-chestnut leaf miner. Catechins seem to be compounds involved in plant resistance. The leaf anatomy showed enhanced accumulation of phenolic compounds at the pest foraging sites. Hypertrophy and thickened and cracked cell walls of the spongy parenchyma were visible in the vicinity of the mines.

## 1. Introduction

Phenolic compounds constitute a broad group of plant secondary metabolites, which are assigned an important role in plant physiology. They are synthesized in all plant organs, and the type and concentration of these compounds depends on their function in the plant. By absorbing UV radiation, flavonoids present in stems and leaves are part of a system protecting the photosynthetic apparatus against the toxicity of this radiation. Flavonoids and anthocyanins are present in flowers and fruits as an attractant for pollinating insects [1]. Phenolic acids predominate in roots and seeds and constitute a chemical protective barrier protecting these tissues against infections. Tannins, which can be present in all plant organs, fulfill a similar function [2,3]. The concentration and profile of phenolic compounds varies not only between plant species but also within one species. Many external factors, including habitat conditions (insolation, soil type, temperature), the growing season, bacterial and viral infections, or the presence of pests, affect their final content. It has been unequivocally confirmed that these compounds constituting the natural protective system of plants are produced with greater intensity in response to stress factors and offer chemical protection against herbivorous organisms [4].

The horse chestnut (*Aesculus hippocastanum* L.) is a park tree common in North America. In Europe, it was spread in the 16th–17th centuries as an attractive ornamental tree. It is characterized by large green leaves and original inflorescences and fruits. Until 1984, the main threat to horse chestnut was posed by chocolate leaf spot caused by the fungus *Guignardia aesculi* and various leaf-foraging insects, after which a new species of insect called *Cameraria ohridella* was found [5]. In the following years, this insect spread throughout Europe. In Poland, the species *C. ohridella* was observed for the first time near Wrocław in 1998 [6]. The pest quickly infested chestnut trees in the south of the country, and now it is found throughout Poland.

Extensive brown-yellow spots on leaves as well as rapid wilting and premature fall of leaves before the end of the growing season are the external symptoms of *C. ohridella* (horse-chestnut leaf miner) foraging [7,8,9]. As a result of infestation by many (5–7) generations of larvae of the pest, the tree is severely weakened, the photosynthetic efficiency declines, the intensity of flowering and the weight and size of fruit and seeds are reduced, and the size and vitality of developing seedlings decrease [10,11,12]. The anatomical and histochemical studies of chestnut leaves conducted to date have shown a complete absence of palisade parenchyma, damaged spongy parenchyma cells, and death of the epidermis on both sides of the leaf blade in the mine, i.e., the pest foraging site [7,8].

The easiest way to reduce the abundance of the leaf beetle is raking and destroying or composting fallen leaves together with chrysalises [13,14,15]. The chemical protection of horse chestnut trees against leaf rot is possible with the use of insecticides sprayed on the crowns or injected directly into trees or into the soil [15]. At the same time, there is a search for natural defensive substances synthesized by the plants to replace synthetic pesticides used for plant protection against the invasion by these insects.

Earlier studies have shown variable sensitivity of different species of the genus *Aesculus* to foraging by the horse-chestnut leaf miner [16]. Similarly, the content of phenolic compounds in healthy and infested leaves of different species varied [16,17]. Therefore, the aim of the present study was to assess changes in the qualitative and quantitative composition of secondary metabolites in horse chestnut leaves collected in different locations and differing in the sensitivity of the plant to the invasion by the horse-chestnut leaf miner and to identify compounds associated with increased plant resistance to this threat. Additionally, changes in the anatomy of chestnut leaves affected by the pest were presented and the sites of accumulation of phenolic compounds in these organs were determined.

## 2. Results and Discussion

The first harvest was carried out when the horse chestnut had fully developed leaves but *C. ohridella* larvae had not yet started foraging. It took place in May and only healthy leaves were collected. The analysis of the chemical composition of the horse chestnut leaves in the initial growing season showed that, depending on the location, the leaves produced different concentrations of secondary metabolites. Among the four analyzed locations, the highest concentration of tannins, phenolic compounds, and flavonoids was recorded for the **O** location. At the same time, the plant was characterized by the highest antiradical activity of the extracts (Table 1). In turn, the leaves from plants growing in the **N** and **S** locations were characterized by the poorest quality parameters. Both phenolic compounds and tannins constitute a group of secondary metabolites that modulate plant resistance to herbivore attack [2,3]. Tannins can defend leaves against insect herbivores by deterrence and/or toxicity. Barbehenn and Constabel [3] noted that tannin toxicity in insects is thought to result from the production of high levels of reactive oxygen species which is closely connected with chemical structure of the molecule. The health condition of plants during the growing season depends on many factors, e.g., genetic and habitat factors, which include soil quality, water supply, and sunlight. As shown in Table 1, the trees of the same species differed in the composition of secondary metabolites already in the initial growing season, which should be attributed to the influence of habitat conditions. The **O** location (the Botanical Garden in Lublin) was the most favorable for plant development based on the antioxidant activity measured. The analyses carried out on subsequent dates were aimed at verifying the thesis that high levels of these compounds are associated with higher plant resistance to the invasion by the horse-chestnut leaf miner and the invasion by *C. ohridella* larvae induces the synthesis of these compounds.

**Figure 1:**
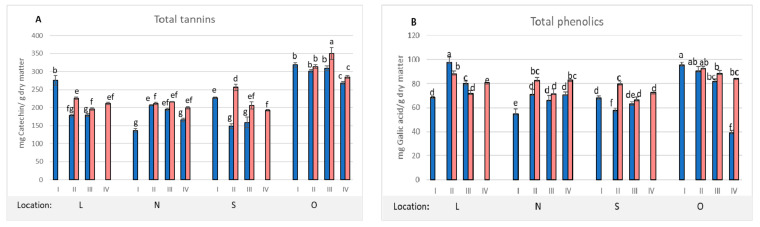

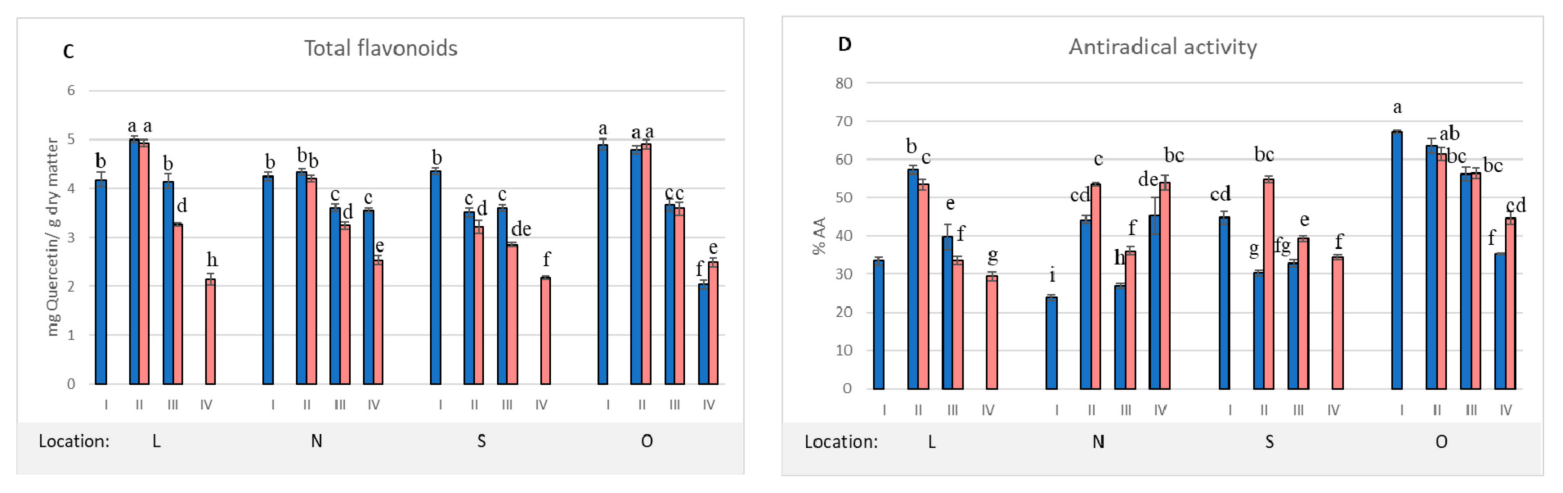
Changes in the content of secondary metabolites and antiradical activity of uninfested (blue) and infested (pink) chestnut leaves at four harvest dates. (**A**) Total tannins; (**B**) Total phenolics; (**C**) Total flavonoids; (**D**) Antiradical activity. The mean values denoted by different letters differ statistically significantly at *p* < 0.05.

**Figure 2:**
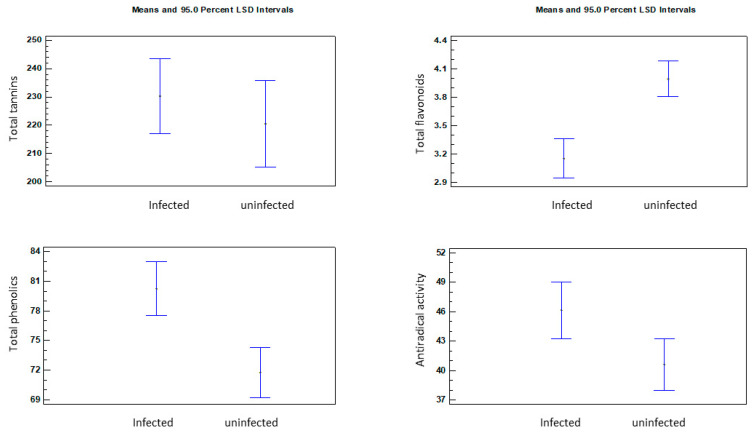
Results of multivariate variance for leaf condition as a factor.

On the subsequent harvest dates (II, III, and IV), the content of secondary metabolites was analyzed separately in completely healthy leaves and in infested leaves. The results are summarized in Figure 1A–D. The analysis of the profile of compounds in healthy and infested horse chestnut leaves showed a clear response of the plant to the stress factor, i.e., the foraging *C. ohridella* larvae. In the case of total tannins (Figure 1A), higher concentrations were recorded in the infested leaves on all harvest dates and in all locations, but these differences were not statistically significant (Figure 2). Significant differences were noted only on the third harvest date in the case of the **O** location. However, the horse chestnut from this location showed the highest total tannin concentration in healthy and infested leaves on all harvest dates. The concentration of total phenolic compounds (Figure 1B) increased or decreased in the infested leaves, depending on the location. In the case of the **L** location, a statistically significant decrease in the concentration of these compounds was found. In turn in the other locations, their concentration was higher in the infested leaves, and, in the majority of cases, the differences were statistically significant. The obtained results confirm previous reports that, in response to the stress factor, the plant begins to synthesize higher concentrations of compounds that may support its chemical protection against pathogens [4]. Tannins and phenolic acids deter insects from foraging, and the higher accumulation of these compounds in leaves from the **O** location resulted in better resistance of the entire plant and a reduction in the number of infested leaves. The obtained results are consistent with previous reports on the role of phenolic compounds and tannins in modulating plant resistance to herbivore attacks [2,3,4].

Contrary to the content of total tannins and phenols, a statistically significant decrease in the concentration of flavonoids in the infested versus healthy leaves was recorded in 3 location (L, N and S), which was particularly evident on the third harvest date (Figure 1C). No differences in the concentration of flavonoids between healthy and infested leaves were found only in the leaves from the **O** location. This may be related to the better overall health condition of this plant. Leaves from the **O** location were characterized by a smaller area of *C. ohridella* foraging, which was eventually reflected by the concentration of flavonoids in the healthy parts of the leaves. The reduction in the concentration of flavonoids in the leaves may also have resulted from the redirection of metabolic pathways towards the biosynthesis of phenolic acids or tannins. Additionally, the intensified solar radiation in the later growing season (dates III and IV) may have had an impact on the partial degradation of flavonoids as compounds protecting the plant photosynthetic apparatus [18]. The last parameter determined in this series of tests was the antiradical activity of extracts obtained from the horse chestnut leaves (Figure 1D). The antiradical activity of extracts reflects the activity of their compounds, which has an impact on plant resistance to stress factors [2,3,4]. The analysis of the total concentration of tannins, phenolic compounds, and flavonoids revealed that the content of total phenolic compounds was the most strongly correlated in Pearson correlation analysis with the antiradical activity (r = 0.9063; Table S1), and the highest activity of the extracts was confirmed in the leaves from the **O** location.

In order to assess the significance of the differences in the concentration of the groups of phenolic compounds and the antiradical activity of horse chestnut leaf extracts, a multifactorial analysis of variance was performed, in which plant location, harvest date, and leaf condition (healthy and infested) were the variables. The results showed that the condition of the leaves was the main factor modifying the chemical composition of the extracts (Figure 2). Healthy leaves contained a significantly higher concentration of total flavonoids. In turn, the infested leaves were characterized by a higher concentration of total phenolic compounds and higher antiradical activity of the extracts. At the same time, the analysis did not confirm any statistically significant differences in the total tannin concentration between the healthy and infested leaves. However, many scientific studies have found a significant effect of the concentration of tannins and phenolic compounds in horse chestnut leaves on the inhibition of the intensity of *C. ohridella* foraging [14,15]. Schultz [19] showed that the chemical composition in leaves of the species *Betula lutea* and *Acer saccharum* influenced the resistance of these plants to damage caused by parasites. Leaves with a higher concentration of tannins were less damaged by pests, compared to leaves with a lower concentration of these compounds. Oszmiański et al. [17] showed a positive correlation between the concentration of polymeric tannins and a higher resistance of trees to the invasion by *C. ohridella* larvae. In turn, Paterska et al. [20] found that the inhibition of larval foraging may result from the increased synthesis of flavonols as defensive compounds. In the present study, despite the lack of significant differences between the concentration of tannins in healthy and infested leaves, the effect of their concentration on the general condition of plants can be noticed. The best parameters were exhibited by the tree from the **O** location, in which the highest total concentration of tannins was recorded from the beginning of the growing season. Interesting results were obtained in the **N** location, where the lowest concentration of tannins was determined in the initial growing season (Figure 1A). On the subsequent harvest dates, an increase in the concentration of these compounds was found, and the condition of the plant was ranked immediately behind that from the **O** location. Therefore, it can be concluded that larval foraging is not a factor inducing the synthesis and accumulation of these compounds in leaves, although they have a direct effect on inhibition of foraging.

**Figure 3:**
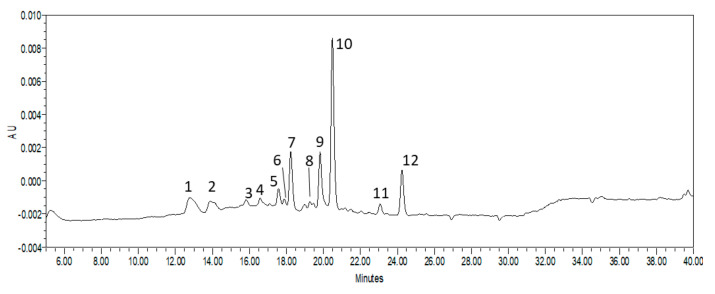
HPLC-DAD chromatogram of horse chestnut leaf extract (1: catechin; 5: Quercetin–3–*O*–galactoside; 6: Quercetin–3–*O*–glucoside; 10: Quercetin–3–*O*–rhamnoside).

The HPLC analysis with DAD detection was carried out for more exact determination of the chemical composition of the extracts from the horse chestnut leaves. The presence of twelve compounds was confirmed in the tested extracts, which were numbered sequentially in the chromatogram from 1 to 12 (Figure 3). Four compounds were identified on the basis of standard compounds; these were catechin (compound **1**), quercetin 3–*O*–galactoside (compound **5**), quercetin 3–*O*–glucoside (compound **6**), and quercetin 3–*O*–rhamnoside (compound **10**). Chromatograms of standards are presented in supplementary material (Figure S1). The analysis of the UV-Vis spectra of the other compounds classified them into two groups: the first one comprised catechins and their derivatives, which in the UV-Vis profile were characterized by one absorption maximum at a wavelength of about λ = 275–280 nm [21]. The second group was composed of flavonols and their derivatives, for which two absorption maxima were observed at about λ = 260 and 350 nm (Figure S2) [21].

**Figure 4:**
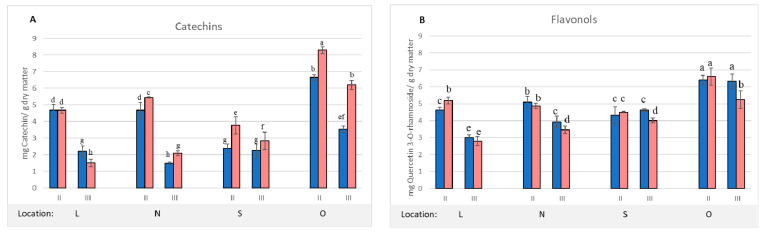
The sum of catechins (**A**) and the sum of flavonols (**B**) in uninfested (blue) and infested (pink) chestnut leaves on two harvest dates, calculated on the basis of HPLC analysis. The mean values denoted by different letters differ statistically significantly at *p* < 0.05.

The first group includes compounds marked in the chromatogram with the numbers: **1**, **2**, **3**, **4**, and **7**. The second group comprises compounds with the numbers: **5**, **6**, **8**, **9**, **10**, **11**, and **12**. For the quantitative analysis of the described compounds, the sum of the compounds of the first group was expressed as the catechin equivalent, and the compounds of the second group were expressed as the equivalent of quercetin 3–*O*–rhamnoside, because this compound was characterized by the highest peak (Figure 3). The changes in the content of the sum of catechins and the sum of flavonols on the two harvest dates are shown in Figure 4.

The Principal Components Analysis (Figure S3) confirmed the observed relationships and clearly indicated that the condition of the leaves and the harvest date were the factors differentiating the concentration of catechins. Their higher concentration was recorded in the infested leaves in practically all locations and dates of harvesting. However, during the growing season, the content of catechins decreased. In contrast, the analysis of flavonols showed different dependencies. Only the location differentiated the concentration of these compounds in the leaves. In the healthy and infested leaves, the differences were usually statistically insignificant (Figure 4 and Figure S4). A number of literature reports confirm the fact that flavonols, along with tannins, constitute the chemical defense of plants against herbivores [18,19]. The present study lists a group of catechins which, in addition to hydrolyzing and non-hydrolyzing tannins, are considered to be the third group of catechin tannins [22,23]. On the basis of the present results, it can be concluded that both the accumulation of these compounds in the initial growing season and the ability to synthesize them in subsequent periods affect the overall condition and resistance of plants.

**Figure 5:**
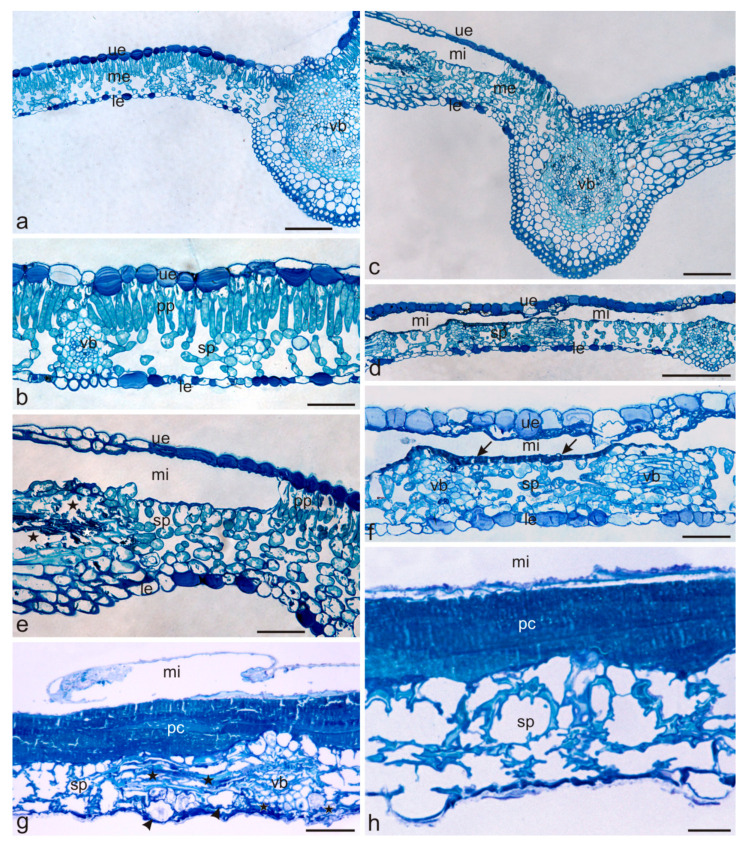
Cross-sections of *Cameraria ohridella*-infested and uninfested *Aesculus hippocastanum* leaves. (**a**,**b**) Uninfested (healthy) leaves with the adaxial (upper) and abaxial (lower) epidermis, mesophyll composed of a palisade and spongy parenchyma, and collateral vascular bundles. (**c**–**f**) Infested (damaged) leaves collected in July. (**c**,**d**) Visible mines at the sites of foraged palisade parenchyma. (**e**) Damaged spongy parenchyma cell walls in the vicinity of the mine (asterisks). (**f**), A thin, dark blue layer containing phenolic compounds visible on the bottom of the mine (arrows). (**g**,**h**) Infested leaves collected in September. (**g**) A substantially thickened amorphous layer containing phenolic compounds visible in the bottom of the mine. Note the cellular hypertrophy (arrows), the thickened walls, and the disturbed cell arrangement of the spongy parenchyma (asterisks). (**h**) Spongy parenchyma cells with cracked walls and a disturbed arrangement visible under the thickened layer containing phenolic compounds. Abbreviations: me—mesophyll, ue—upper epidermis, le—lower epidermis, pp—palisade parenchyma, sp—spongy parenchyma, vb—vascular bundles, mi—mines, pc—phenolic compounds. Scale bars: 200 µm (**d**), 100 µm (**a**,**c**), 50 µm (**b**,**e**–**g**), 20 µm (**h**).

The cross-sections through the healthy chestnut leaf blade of showed the adaxial (upper) epidermis, one layer of palisade parenchyma, several layers of spongy parenchyma with collateral vascular bundles surrounded by a sclerenchymatous sheath, and the abaxial (lower) epidermis with stomata (Figure 5a,b). In the healthy and damaged leaves, the cells of the adaxial epidermis and, to a lesser extent, the abaxial epidermis contained phenolic compounds, which stained intensely with Toluidine blue O (Figure 5a–f).

In the infested leaves (collected in July), the yellow-brown spots with the mines exhibited large empty spaces with foraged palisade parenchyma and a thin dark blue layer of phenolic compounds on the bottom of the mines adjacent to the spongy parenchyma (Figure 5e,f). In many areas, the cells of the spongy parenchyma and cells adjacent to the vascular elements were destroyed, which was manifested by damaged cell walls and a disturbed arrangement of the cells (Figure 5e,f). In the second term (September), the foraging symptoms intensified. The infested leaves exhibited a detached and degenerated adaxial epidermis layer, hypertrophy, thickened and cracked cell walls of spongy parenchyma, and a several-fold increase in the thickness of the amorphous layer on the bottom of the mine containing phenolic compounds (Figure 5g,h). The abaxial epidermis cells were destroyed as well (Figure 5g,h). As reported by Padayachee et al. [24], the production of thicker cell walls is one of the strategies of plant defence against *C. ohridella* infestation.

**Figure 6:**
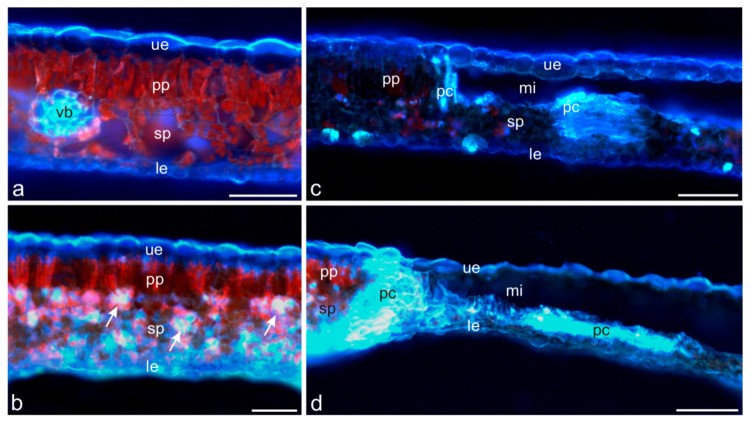
Light blue autofluorescence of phenolic compounds in *Camararia ohridella*-uninfested (**a**) and infested (**b**–**d**) *Aesculus hippocastanum* leaf tissues. (**a**) Autofluorescence of phenolic compounds visible in the adaxial epidermis and in the vascular bundle cell walls. (**b**,**c**) Infested leaves collected in July: autofluorescence of phenolic compounds visible in the adaxial and abaxial epidermis and in the spongy parenchyma (arrows) (**b**) and in tissues adjacent to the pest foraging site (**c**). (**d**) Infested leaves collected in September: autofluorescence of phenolic compounds visible on the bottom of the mines and in the palisade and spongy parenchyma cells adjacent to the mine. Abbreviations: ue—upper epidermis, le—lower epidermis, pp—palisade parenchyma, sp—spongy parenchyma, vb—vascular bundles, mi—mines, pc—phenolic compounds. Scale bars: 100 µm (**c**,**d**), 50 µm (**a**,**b**).

The fluorescence microscopy observations indicated an increased amount of phenolic compounds in the horse chestnut leaves colonised by *C. ohridella*. In the healthy leaves, the light blue autofluorescence of phenolic compounds was mainly visible in the outer cell walls of the adaxial epidermis and in the cell walls of the vascular bundles (Figure 6a). In turn, phenolic compounds in theleaves damaged by the foraging *C. ohridella* larvae (July) were present in not only adaxial but also abaxial epidermis cells and in spongy parenchyma cells (Figure 6b). They accumulated mainly in tissues adjacent to the pest foraging sites (Figure 6c).

In the case of the larger mines (September), substantial amounts of fluorescent phenolic compounds were present at the border of mines with the healthy palisade and spongy parenchyma and in their lower parts (Figure 6d). Our present and previous studies [25] have demonstrated that *C. ohridella* larvae infesting horse chestnut leaves feed mainly on the palisade parenchyma, which is characterized by a compact arrangement of cells and large numbers of chloroplasts. As shown by literature data, the leaf mesophyll containing numerous chloroplasts is the principal specialized photosynthetic tissue in plants [26]. The palisade parenchyma is characterized by a high surface-to-volume ratio, which facilitates CO_2_ absorption in this leaf area exposed to high amounts of light and ensures a high photosynthesis rate [27,28]. Since the synthesis of organic compounds is more efficient in the palisade parenchyma than in the spongy parenchyma, larvae of the pest find a rich source of food and ingest mainly the former layer.

In our histochemical study [25] and in the current research, we have analyzed semi-thin preparations and used the fluorescence method to show that phenolic compounds in healthy *A. hippocastanum* leaves are present only in the epidermis on both sides of the leaf blade. As reported in the literature, the vacuoles of epidermis cells accumulate many soluble phenolic compounds: flavonoids [29,30], phenolic acids [31], anthocyanins [32], and tannins [25,33]. Phenolic acids and flavonoids may be present in the cuticle [34], and flavonoids and tannins may be associated with epidermis cell walls [35]. The content of phenolic compounds in superficial tissues is the first line of defense against pathogens and insects. Flavonol aglycones, flavone aglycones, flavanone aglycones, catechins, and tannins have been reported to be highly toxic to herbivores and pathogens [30].

Using the same methods to analyze the healthy leaves, we found no phenolic compounds in the palisade and spongy parenchyma. In turn, phenolic compounds were accumulated also in the spongy parenchyma in leaves colonised by *C. ohridella* larvae. Phenolic substances can affect the taste of tissues and have a toxic effect [36]. This may be another cause of the choice of the palisade parenchyma by *C. ohridella* larvae. Phenolic compounds in parenchyma cells are accumulated in different subcellular compartments. They are mainly distributed in the vacuole and the cell wall. The vacuole mainly accumulates soluble phenolics, e.g., simple phenols, flavonoids, and tannins [37], while the cell wall contains primarily phenolic acids [30]. Moreover, phenolic compounds have been detected in the cell nucleus [38], chloroplasts, and mitochondria [39].

The present anatomical studies confirm and supplement the results of phytochemical studies indicating increased content of phenolic compounds in horse chestnut leaves colonized by *C. ohridella*.

## 3. Materials and Methods

### 3.1. Plant Material and Extract Preparation

Chestnut leaves collected in four periods of one growing season in 2020: I: 10 May, II: 10 June, III: 10 August, and IV: 10 September were the plant material. The leaves were collected randomly from four trees located in different locations in Lublin, Poland (51°16′ N, 22°34′ E), which were symbolically marked with the letters **L**, **N**, **O,** and **S**. The **L and N** locations were trees growing in greenery on city squares. The **S** location was represented by a tree growing on a fairly busy street, while the tree from the **O** location was growing in the Botanical Garden in Lublin. Each time, 50 leaves were collected from each location from trees of different height (from 1 to 3 m). Completely healthy (u-i: uninfested) and infested (i: infested) leaves were collected separately. The dry matter content was determined with the drying method in healthy leaves without petioles.

The leaves from the first and subsequent harvests were dried at a temperature of 35 °C for seven days and stored in a dry, cool, and dark place. Before the analysis, samples of raw materials were shredded, and weighed leaf samples (0.1 g) were homogenized in 90% aqueous methanol (50 mL) with ultrasound assisted static extraction (2 × 9 min). The extract was filtered through a filter paper (Whatman no. 42) after 30 min and used for analysis.

### 3.2. Total Tannins (TT)

Tannins were determined as described by Price et al. [40] with minor modifications proposed by Osman [41]. The plant sample (1.0 g) was mixed with 10 mL of a 1% HCl in methanol in a dark bottle and shaken for 24 h at room temperature. Then, the mixture was filtrated. The tannins in the supernatant were estimated using 1 mL of the supernatant and 5 mL of a vanillin/HCl mixture (by mixing equal volumes of 2% vanillin in methanol and 8% HCl in methanol) in a test tube and kept for 20 min at room temperature. The color formed was determined at 500 nm. Catechin (Ctch) was used to prepare the standard curve and the content of total tannins (TT) was expressed as catechin equivalents (mg catechin/g dry matter).

### 3.3. Total Phenolics (Folin)

The total content of phenolic compounds was determined with the Folin–Ciocalteu reagent method [42,43]. The reaction mixture consisted of the following reagents: 60 µL of the tested extract, 1.5 mL of Folin’s reagent diluted in water in a ratio of 1:10, 1.2 mL of sodium bicarbonate (75 g/L), and 0.54 mL of distilled water. The reaction mixture was stored at room temperature for 30 min. Absorbance was measured at a wavelength of λ = 760 nm. The sum of phenolic compounds was expressed as gallic acid equivalents [mg gallic acid/g dry matter].

### 3.4. Total Flavonoids (TF)

The sum of flavonoids contained in the analyzed extracts was determined with the AlCl_3_ method [44]. The reaction mixture consisted of the following reagents: 0.5 mL of the tested extract, 1.5 mL of ethanol 96%, 0.1 mL of AlCl_3_ (10%), 0.1 mL of sodium acetate (1M), and 2.8 mL of distilled water. The reaction mixture was stored at room temperature for 40 min and the absorbance was measured at a wavelength λ = 415 nm. The sum of flavonoids was expressed as quercetin equivalents [mg quercetin/g dry matter].

### 3.5. Antiradical Activity (AA)

The antiradical activity was determined in the system with the 1,1-diphenyl-2-picrylhydrazyl radical (DPPH^•^) according to the method proposed by Conforti et al. [45]. The reaction mixture consisted of the following reagents: 100µLof extract and 4 mL of a 0.1 mM DPPH solution. The sample was stored at room temperature for 30 min, and then the absorbance was measured at λ = 515 nm. The antioxidant activity (AA) expressed as a percentage of reduction of DPPH radicals was calculated from the following formula:

%AA= [1 − (Ap/Ab)] × 100%(1)

AA—antioxidant activity of the analyzed sample
Ap—absorbance of the analyzed sample
Ab—absorbance of the blank sample

### 3.6. HPLC Analysis

The HPLC method was used for detailed investigations of the chemical profile of the extracts from the infested and uninfested leaves. The analysis was performed using an Empower-Pro chromatograph (Waters), which consists of a quaternary pump (M2998 Waters) with a degasser and a UV-Vis diode array detection (DAD) system. Separation was performed on a column filled with modified silica gel RP-18 (Atlantis T3—Waters, 3 μm, 4.6 mm × 150 mm). The mobile phase consisted of A (1% acetic acid in water) and B (acetonitrile). The program of the gradient elution was set as follows: 20% B (0 to 10 min); 20–25% B (10 to 25 min); 25–45% B (25 to 40 min); the flow speed was 1 mL/min. The qualitative analysis of phenolic compounds was performed on the basis of retention times and diode array spectral characteristics in comparison with available standards of phenolic compounds. In addition, quantitative investigations were performed on the basis of the areas of the peaks of the tested compounds and calibration curves prepared separately for each standard compound. The DAD detection was conducted at 280 nm for phenolic acid derivatives and at 330 nm for flavonoid derivatives for the purpose of quantitative analysis. The content of phenolic derivatives was expressed as mg/g of dry matter.

### 3.7. Microscopic Analysis

Leaves for microscopic examinations were collected in two terms: I (July) and II (September). Fragments of healthy (uninfested) leaves (*n* = 10 in each term) and infested leaves (*n* = 10 in each term) were taken from the central part with the midrib of one of the leaflets forming the compound leaf. They were fixed in 2.5% glutaraldehyde in 0.1 M phosphate buffer at pH 7.2 for 12 h at 4 °C. Next, they were washed three times in phosphate buffer (pH 7.2), dehydrated in an ethanol series, and embedded in LR white resin (LR white acrylic resin, medium grade, Sigma-Aldrich). Semi-thin cross-sections (0.7 μm thick) were cut with glass knives using a Reichert Ultracut S ultramicrotome and stained with 1% aqueous Toluidyne blue O [46]. The sections were examined and imaged under a Nikon SE 102 light microscope (Nikon, Japan). Additionally, cross-sections of fresh unfixed and unstained fragments of uninfested and infested leaves (*n* = 5 for each period) cut with a razor blade were embedded in water with glycerol (1:1) and examined by means of fluorescence microscopy to determine the location of phenolic acids [21,47]. Light blue autofluorescence of phenolic acids excited with UV radiation was observed by means of a Nikon Eclipse 90i microscope (Tokyo, Japan) with a digital camera (Nikon Fi1) and NIS-Elements Br 2 using a Cy5 filter (excitation wavelength 590–650 nm) and a barrier filter (wavelength 663–738 nm).

### 3.8. Statistical Analysis

The results were performed in three replications, and the data were expressed as the mean ± SD. The significance of differences between the means was determined with LSD multi-way ANOVA test with 5% error probability. To assess the relationship between the chemical content and the antiradical activity of the analyzed extracts, Pearson’s correlation analysis was performed. A *p*-value <0.01 was considered to be significant. Statistical comparisons were performed using the Statgraphics Centurion software, version XVI (Statgraphics Technologies Inc., The Plains, VA, USA). In addition, a Principal Component Analysis (PCA) was performed for compounds determined with the HPLC method. For this purpose, Statistica software was used (version 13.1 PL; StatSoft Inc., Tulsa, OK, USA; StatSoft, Krakow, Poland).

## 4. Conclusions

The obtained results confirm that, as part of secondary metabolism, plants are able to intensify the synthesis of compounds that constitute a form of defense against pest invasion, i.e., phenolic compounds, mainly catechins. The fluorescence technique used in the analysis of leaf tissues helped to localize areas of remarkable accumulation of phenols. We have shown that phenolic compounds accumulate mainly in tissues adjacent to the foraging sites and below mines produced by larvae, which indicates a clear defense response of the plant and confirms some literature reports [48]. It was also found that the good condition of the plant, manifested by high concentrations of secondary metabolites already in the early growing season, allows the plant to limit herbivore infestations and extend the period of good leaf condition. Catechin compounds appear to be related to plant resistance, but further research is needed to identify these substances within the layer surrounding the mines.

## Figures and Tables

**Table 1:** Content of secondary metabolites in healthy leaves on the first harvest date.

Location	Dry Matter (%)	Total Tannins ^1^	Total Phenolics ^2^	Total Flavonoids ^3^	Antioxidant Activity ^4^
L	70.7 ^b^ ± 0.2	275.1 ^a^ ± 26.9	68.5 ^b^ ±1.4	4.2 ^b^ ± 0.3	33.4 ^c^ ± 2.1
N	69.8 ^b^ ± 0.9	136.6 ^c^ ± 10.4	54.9 ^c^ ± 8.2	4.2 ^b^ ± 0.2	23.9 ^d^ ± 1.8
S	78.7 ^a^ ± 0.3	217.4 ^b^ ± 4.2	67.9 ^b^ ± 3.3	4.4 ^b^ ± 0.1	44.7 ^b^ ± 3.3
O	67.5 ^bc^ ± 0.2	318.9 ^a^ ± 12.6	95.6 ^a^ ± 4.5	4.9 ^a^ ± 0.2	67.3 ^a^ ± 1.1

^1^ mg catechin/g dry matter; ^2^ mg gallic acid/g dry matter; ^3^ mg quercetin/g dry matter; ^4^ %AA. The values are expressed as the mean ± SD (*n* = 3). Different letters in the same column indicate significant differences between the means at *p* < 0.05 in the LSD one-way ANOVA test.

## Data Availability

Not applicable.

## Supplementary Materials

The following supporting information can be downloaded at: https://www.mdpi.com/article/10.3390/molecules27175471/s1, Figure S1. Chromatograms of standard compounds. Figure S2: UV-Vis spectra from HPLC-DAD analysis of phenolic compounds quantified in extracts of chestnut leaves. Figure S3. Principal Components Analysis for catechins identified by HPLC. Figure S4. Principal Components Analysis for flavonols identified by HPLC. Table S1. Pearson’s correlation coefficients.

## References

[1] Ordano M., Blendinger P.G., Lomáscolo S.B., Chacoff N.P., Sánchez M.S., Montellano M.G.N., Jiménez J., Ruggera R.A., Valoy M. (2017). The role of trait combination in the conspicuousness of fruit display among bird-dispersed plants. Funct. Ecol..

[2] Barbehenn R.V., Constabel C.P. (2011). Tannins in plant–herbivore interactions. Phytochemistry.

[3] Gourlay G., Constabel C.P. (2019). Condensed tannins are inducible antioxidants and protect hybrid poplar against oxidative stress. Tree Physiol..

[4] Bennett R.N., Wallsgrove R.M. (1994). Secondary metabolites in plant defence mechanisms. New Phytol..

[5] Deschka G., Dimic N. (1986). *Cameraria ohridella* sp.n. (*Lep., Lithocolletidae*) from Macedonia, Yugoslavia. Acta Entomol. Jugosl..

[6] Łabanowski G., Sojka G. (1998). Szrotówek kasztanowcowiaczek zagraża kasztanowcom w Polsce. Ochr. Roślin.

[7] Weryszko-Chmielewska E., Haratym W. (2011). Changes in leaf tissues of common horse chestnut (*Aesculus hippocastanum* L.) colonised by the horse-chestnut leaf miner (*Cameraria ochridella* Deschka and Dimić). Acta Agrobot..

[8] Weryszko-Chmielewska E., Haratym W. (2012). Leaf micromorphology of *Aesculus hippocastanum* L. and damage caused by leaf-mining larvae of Cameraria ohridella Deschka and Dimić. Acta Agrobot..

[9] Myśkow E., Sokołowska K., Słupianek A., Gryc V. (2021). Description of Intra-Annual Changes in Cambial Activity and Differentiation of Secondary Conductive Tissues of *Aesculus hippocastanum* Trees Affected by the Leaf Miner *Cameraria ohridella*. Forests.

[10] Percival G.C., Barrow I., Noviss K., Keary I., Pennington P. (2011). The impact of horse chestnut leaf miner (Cameraria ohridella Deschka and Dimic; HCLM) on vitality, growth and reproduction of *Aesculus hippocastanum* L.. Urban For. Urban Green..

[11] Holoborodko K.K., Seliutina O.V., Ivanko I.A., Alexeyeva A.A., Shulman M.V., Pakhomov O.Y. (2021). Effect of *Cameraria ohridella* feeding on *Aesculus hippocastanum* photosynthesis. Regul. Mech. Biosyst..

[12] Tyapkina A.P., Silaeva Z.G., Kiseleva L.L., Shiryaeva N.A., Parakhina E.A. Ecological characteristics of horse chestnut (*Aesculus hippocastanum* L.) plantings of the city of Orel. Proceedings of the BIO Web of Conferences.

[13] Arnold C., Sengonca C. (2002). Bedeutung von gängigen gartenbaulichen Maßnahmen für die Reduktion des Befallsdrucks der Rosskastanien-Miniermotte *Cameraria ohridella* Deschka & Dimic (Lep., Gracillariidae). Gesunde Pflanz..

[14] Pavan F., Barro P., Bernardinelli I., Gambon N., Zandigiacomo P. (2003). Cultural control of *Cameraria ohridella* on horsechestnut in urban areas by removing fallen leaves in autumn. Arboric. Urban For..

[15] Głowacka B., Lipiński S., Tarwacki G. (2009). Possibilities of protection of horse chestnut *Aesculus hippocastanum* L. against horse chestnut leaf-miner *Cameraria ohridella* Deschka et Dimić. For. Res. Pap..

[16] Oszmiański J., Kalisz S., Wojdyło A. (2014). The content of phenolic compounds in leaf tissues of white (*Aesculus hippocastanum* L.) and red horse chestnut (*Aesculus carea* H.) colonized by the horse chestnut leaf miner (*Cameraria ohridella* Deschka & Dimić). Molecules.

[17] Oszmiański J., Kolniak-Ostek J., Biernat A. (2015). The content of phenolic compounds in leaf tissues of *Aesculus glabra* and *Aesculus parviflora* Walt. Molecules.

[18] Ferreyra M.L.F., Rius S.P., Casati P. (2012). Flavonoids: Biosynthesis, biological functions, and biotechnological applications. Front. Plant Sci..

[19] Schultz J.C., Hein P.A. (1983). Impact of Variable Plant Defensive Chemistry on Susceptibility of Insects to Natural Enemies.

[20] Paterska M., Bandurska H., Wysłouch J., Molińska-Glura M., Moliński K. (2017). Chemical composition of horse-chestnut (*Aesculus*) leaves and their susceptibility to chestnut leaf miner *Cameraria ohridella* Deschka & Dimić. Acta Physiol. Plant..

[21] Mabry T.J., Markham K.R., Thomas M.B. (1970). The Systematic Identification of Flavonoids.

[22] Okuda T., Ito H. (2011). Tannins of Constant Structure in Medicinal and Food Plants—Hydrolyzable Tannins and Polyphenols Related to Tannins. Molecules.

[23] Pleszczyńska M., Szczodrak J. (2005). Taniny i ich rozkład enzymatyczny. Biotechnologia.

[24] Padayachee A., Netzelb G., Netzelb M., Day L., Zabarase D., Mikkelsena D., Gidleya M.J. (2012). Binding of polyphenols to plant cell wall analogues—Part 2: Phenolic acids. Food Chem..

[25] Konarska A., Grochowska M., Haratym H., Tietze M., Weryszko-Chmielewska E., Lechowski L. (2020). Changes in *Aesculus hippocastanum* leaves during development of *Cameraria ohridella*. Urban For. Urban Green..

[26] Evert R.F. (2006). Esau’s Plant Anatomy: Meristems, Cells, and Tissues of the Plant Body: Their Structure, Function and Developmnent.

[27] Théroux-Rancourt G., Roddy A.B., Earles J.M., Gilbert M.E., Zwieniecki M.A., Boyce C.K., Tholen D., McElrone A.J., Simonin K.A., Brodersen C.R. (2021). Maximum CO_2_ diffusion inside leaves is limited by the scaling of cell size and genome size. Proc. R. Soc. B Boil. Sci..

[28] Borsuk A.M., Brodersen C.R. (2019). The spatial distribution of chlorophyll in leaves. Plant Physiol..

[29] Dmitruk M., Sulborska A., Żuraw B., Stawiarz E., Weryszko-Chmielewska E. (2019). Sites of secretion of bioactive compounds in leaves of *Dracocephalum moldavica* L.: Anatomical, histochemical, and essential oil study. Braz. J. Bot..

[30] Karabourniotis G., Liakopoulos G., Nikolopoulos D., Bresta P., Stavroulaki V., Sumbele S. (2014). “Carbon gain vs. water saving, growth vs. defence”: Two dilemmas with soluble phenolics as a joker. Plant Sci..

[31] Cerovic Z.G., Ounis A., Cartelat A., Latouche G., Goulas Y., Meyer S., Moya I. (2002). The use of chlorophyll fluorescence excitation spectra for the non-destructive in situ assessment of UV-absorbing compounds in leaves. Plant Cell Environ..

[32] Manetas Y. (2006). Why some leaves are anthocyanic and why most anthocyanic leaves are red?. Flora Morphol. Distrib. Funct. Ecol. Plants.

[33] Chernetskyy M., Woźniak A., Skalska-Kamińska A., Żuraw B., Blicharska E., Rejdak R., Donica H., Weryszko-Chmielewska E. (2018). Structure of leaves and phenolic acids in *Kalanchoë daigremontiana* Raym.-Hamet & H. Perrier. Acta Sci. Pol. Hortorum Cultus.

[34] Karabourniotis G., Liakopoulos G. (2005). Phenolic compounds in plant cuticules: Physiological and ecological aspects. Adv. Plant Physiol..

[35] Kariotia A., Bilia A.R., Skaltsa H. (2010). *Quercus ilex* L.: A rich source of polyacylated flavonoid glucosides. Food Chem..

[36] Pagare S., Bhatia M., Tripathi N., Pagare S., Bansal Y.K. (2015). Secondary metabolites of plants and their role: Overview. Curr. Trends Biotechnol. Pharm..

[37] Ferreres F., Figueiredo R., Bettencourt S., Carqueijeiro I., Oliveira J., Gil-Izquierdo A., Pereira D.M., Valentão P., Andrade P.B., Duarte P. (2011). Identification of phenolic compounds in isolated vacuoles of the medicinal plant *Catharanthus roseus* and their interaction with vacuolar class III peroxidase: An H_2_O_2_ affair?. J. Exp. Bot..

[38] Feucht W., Treutter D., Polster J. (2004). Flavanol binding of nuclei from tree species. Plant Cell Rep..

[39] Agati G., Brunetti C., Di Ferdinando M., Ferrini F., Pollastri S., Tattini M. (2013). Functional roles of flavonoids in photoprotection: New evidence, lessons from the past. Plant Physiol. Biochem..

[40] Price M.L., Socoyoc S.V., Butler L.G. (1978). A critical evaluation of vanillin reaction as an assay for tannin in sorghum grain. J. Agric. Food Chem..

[41] Osman M.A. (2004). Changes in sorghum enzyme inhibitors, phytic acid, tannins and in vitro protein digestibility occurring during Khamir (local bread) fermentation. Food Chem..

[42] Burda S., Oleszek W. (2001). Antioxidant and Antiradical Activities of Flavonoids. J. Agric. Food Chem..

[43] Zheng W., Wang S. (2001). Antioxidant activity and phenolic compounds in selected herbs. J. Agric. Food Chem..

[44] Zhisten J., Mengcheng T., Jianming W. (1999). The determination of flavonoid contents in mulberry and their scavenging effects on superoxide radicals. Food Chem..

[45] Conforti F., Statti G.A., Menichini F. (2007). Chemical and biological variability of hot pepper fruits (*Capsicum annuum* var. acuminatum L.) in relation to maturity stage. Food Chem..

[46] O’Brien T.P., McCully M.E. (1981). The Study of Plant Structure: Principles and Selected Methods.

[47] Harris P.J., Hartley R.D. (1978). Detection of bound ferulic acid in cell walls of the *Gramineae* by ultraviolet fluorescence microscopy. Nature.

[48] De Pinna G.F.A.M., Kraus J.E., De Menezes N.L. (2002). Morphology and anatomy of leaf mine in *Richterago riparia* Roque (Asteraceae) in the campos rupestres of Serra do Cipó, Brazil. Braz. J. Biol..

