# Supplementary material for: The Secondary Metabolites Profile in Horse Chestnut Leaves Infested with Horse-Chestnut Leaf Miner
Source: Molecules. 2022 Aug 25;27(17):5471. doi: 10.3390/molecules27175471 (PMC9458045; doi:10.3390/molecules27175471)
Supplement: Supplementary file 1 [file molecules-27-05471-s001.zip › molecules-1861203-supplementary.pdf]

molecules

MDPI

Supplementary Materials

The Secondary Metabolites Profile in Horse Chestnut Leaves Infested with Horse-Chestnut Leaf Miner

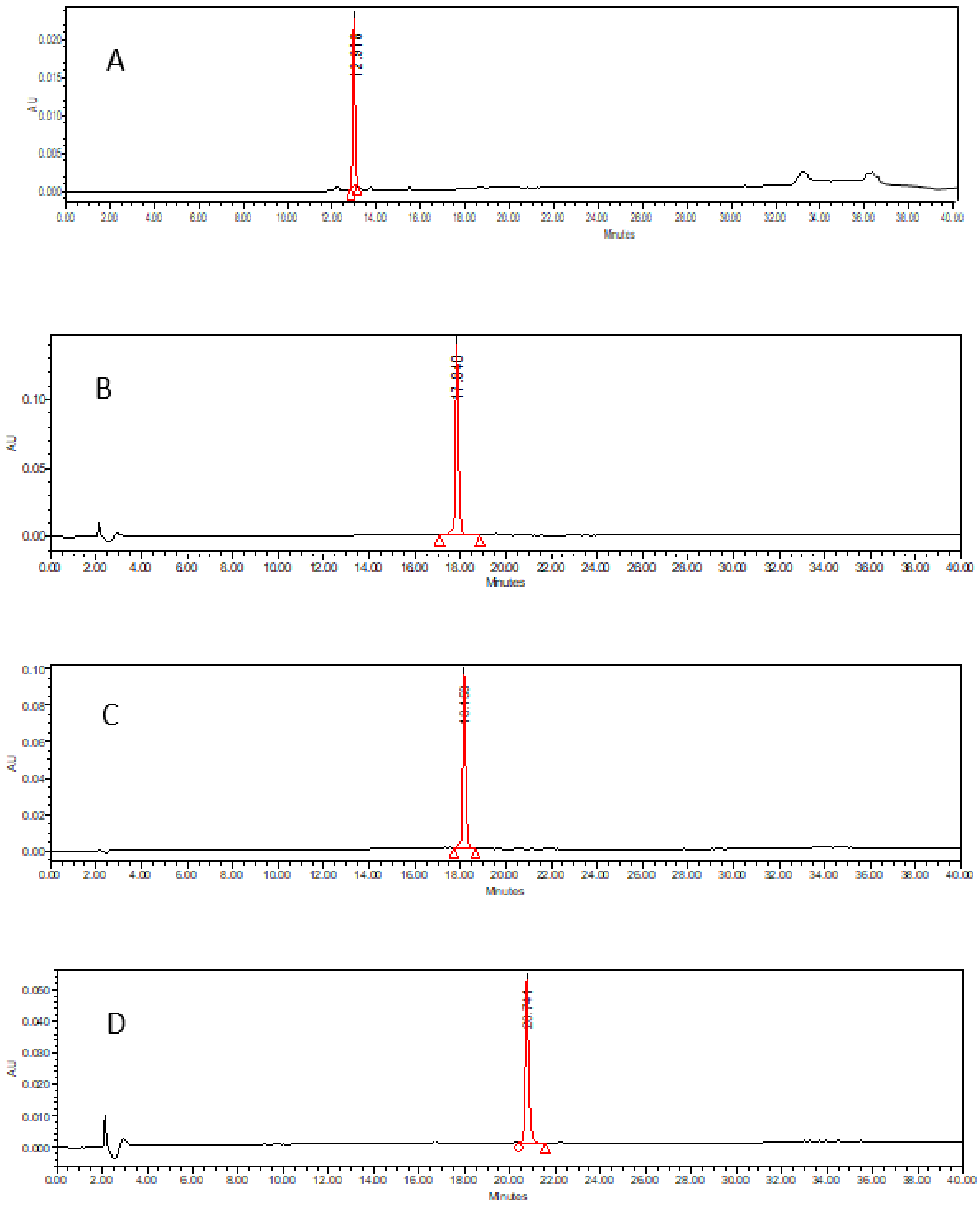

Figure S1. Chromatograms of standard compounds, A: catechin; B: Quercetin-3-*O*-galactoside; C: Quercetin-3-*O*-glucoside; D: Quercetin-3-*O*-rhamnoside.

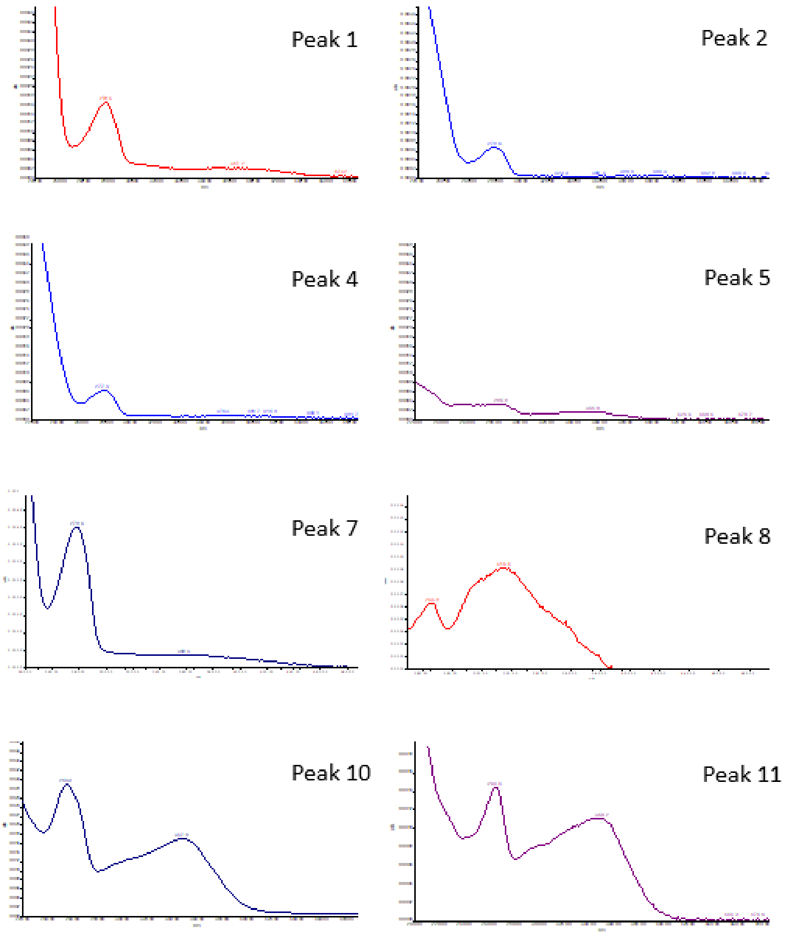

Figure S2. UV-Vis spectra from HPLC-DAD analysis of phenolic compounds quantified in extracts of chestnut leaves.

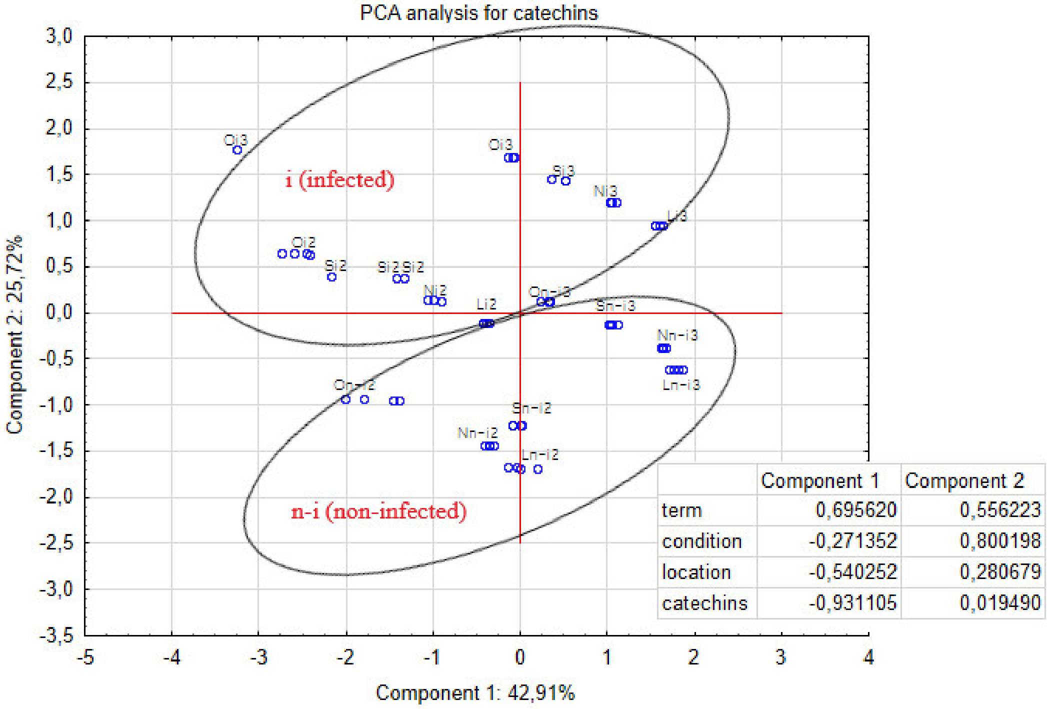

	Component 1	Component 2
term	0,695620	0,556223
condition	-0,271352	0,800198
location	-0,540252	0,280679
catechins	-0,931105	0,019490

Figure S3. Principal Components Analysis for catechins identified by HPLC.

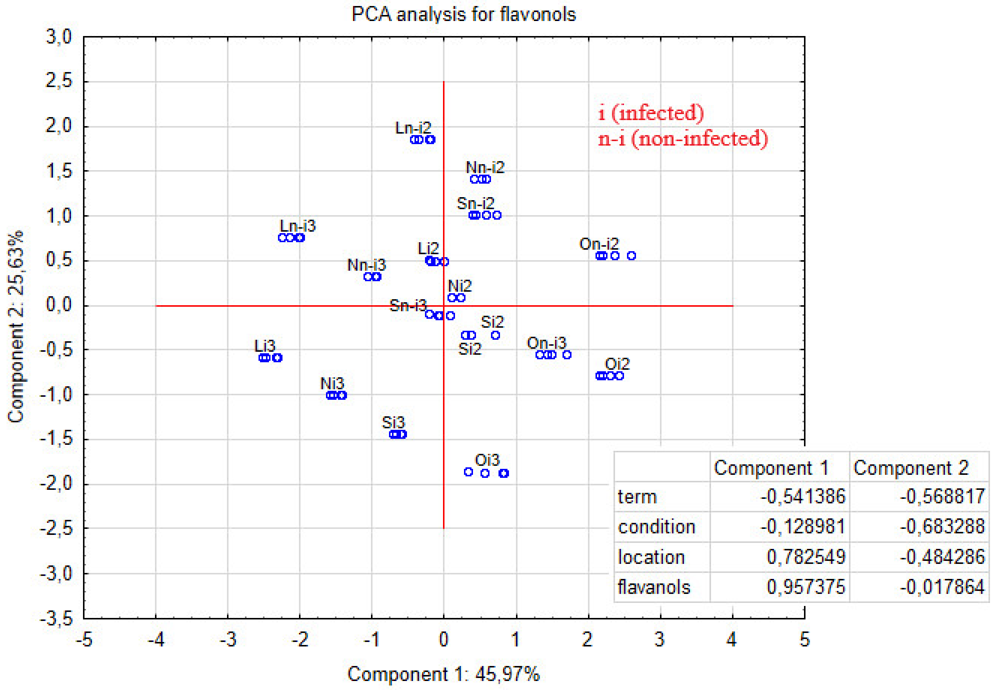

	Component 1	Component 2
term	-0,541386	-0,568817
condition	-0,128981	-0,683288
location	0,782549	-0,484286
flavanols	0,957375	-0,017864

Figure S4. Principal Components Analysis for flavonols identified by HPLC.

Table S1. Pearson's correlation coefficients.

	**Total Tannins**	**Total Phenolics**	**Total Flavonoids**
Antiradical activity	0.711618	**0.90631**	0.642451
Total tannins	1	0.61943	0.350497
Total phenolics		1	0.540493
Total flavonoids			1
